# Effects of Thoracic Mobilization and Extension Exercise on Thoracic Alignment and Shoulder Function in Patients with Subacromial Impingement Syndrome: A Randomized Controlled Pilot Study

**DOI:** 10.3390/healthcare8030316

**Published:** 2020-09-02

**Authors:** Shin Jun Park, Seok Hyeon Kim, Soon Hee Kim

**Affiliations:** 1 Department of Physical Therapy, Gangdong University, 278, Daehak-gil, Gamgok-myeon, Eumseong-gun, Chungcheongbuk-do 27600, Korea; p3178310@gangdong.ac.kr; 2 Department of Physical Therapy, General Graduate School, Yongin University, 134, Yongindaehak-ro, Cheoin-gu, Yongin-si, Gyeonggi-do 17092, Korea; 201851402@yiu.ac.kr; 3 Department of Physical Therapy, Yongin University, 134, Yongindaehak-ro, Cheoin-gu, Yongin-si, Gyeonggi-do 17092, Korea

**Keywords:** joint mobilization, shoulder, trunk extension, SIS

## Abstract

*Introduction:* Thoracic kyphosis commonly occurs in subacromial impingement syndrome. This pilot study investigated the effect of thoracic joint mobilization and extension exercise on improving thoracic alignment and shoulder function. *Methods:* In total, 30 patients with subacromial impingement syndrome were recruited and randomly assigned to three groups, the joint mobilization group (*n* = 10), exercise group (*n* = 10), and combination group (*n* = 10). After four weeks of treatment, the measured outcomes included thoracic kyphosis using a manual inclinometer; pectoralis major (PM) and upper trapezius (UT) muscle tone and stiffness using the MyotonPRO^®^; affected side passive range of motion (ROM) using the goniometer (flexion, abduction, medial rotation, and lateral rotation); and shoulder pain and disability index (SPADI). *Results:* All three groups had significant improvements in all variables (*p* < 0.05). Thoracic kyphosis; UT muscle tone; and flexion, medial rotation, and lateral rotation ROM and SPADI were all significantly improved in the combination group compared to the mobilization and exercise groups (*p* < 0.05). *Conclusions:* The combination therapy of thoracic mobilization and extension exercise can be regarded as a promising method to improve thoracic alignment and shoulder function in patients with subacromial impingement syndrome.

## 1. Introduction

Subacromial impingement syndrome appears as the mechanical block, repeated compressive of the rotator cuff tendons as they pass through the subacromial space; these can result in problems with shoulder functions [1]. Generally, thoracic kyphosis is present in the posture of patients with subacromial impingement syndrome (SIS) [2]. Thoracic kyphosis can be directly or indirectly caused by the development of SIS, indicating scapular dyskinesis [3,4]. Thoracic kyphosis is defined as an anteriorly tilted, downwardly rotated, and protracted scapular posture and indicates a decrease in elevation of the glenohumeral joint in a patient with SIS [2]. As thoracic kyphosis can affect complex shoulder functions, treatments for a neutral thoracic spine can improve shoulder pain and function in patients with SIS [5].

Thoracic extension posture can increase the shoulder active range of motion (ROM) in patients with SIS [6]. Manual therapy focused on thoracic extension mobility has also been shown to improve shoulder function, pain, and kyphotic curvature in patients with SIS [5,7]. Joint mobilization is a type of manual therapy that facilitates movement recovery by inducing minute manual oscillations in the joint hypomobility vertebral segment [8]. Assessments using dynamic magnetic resonance imaging (MRI) have demonstrated the possibility of using joint mobilization to increase spine extension [9]. Thoracic joint mobilization has been shown to increase ROM, minimize pain, and improve shoulder function in patients with SIS through thoracic extension [10]. Nonetheless, manual therapy is a type of passive intervention performed by a physiotherapist [8]. As part of the manual therapy, spine manipulation (high-velocity and low-amplitude) immediately decreased self-reported pain for shoulder movements and SPADI [7,11].

As thoracic kyphosis manifests as an issue not only in noncontractile tissue responsible for maintaining the structure of the thoracic spine but, also, in contractile tissue responsible for facilitating movement [12], active therapeutic exercise is required [13]. An extension-based trunk exercise is a training protocol that can improve thoracic kyphosis [14,15]. A thoracic extension exercise is a correctional exercise that improves the thoracic extensor muscle strength and chest muscle stretch to maintain an optimal postural kinetic chain [16,17]. Thoracic correction exercise also improves kyphosis, pain, and scapular forward distance [14].

Accordingly, active therapeutic exercise could be used as an approach to improve thoracic kyphosis in patients with SIS, along with passive manual therapy performed by a physiotherapist [7,14]. To improve shoulder functions in patients with SIS, exercise with thoracic joint mobilization should be performed, rather than exercise alone [18]. Nevertheless, there remains a lack of research on the individual effects of extension exercise, joint mobilization, and combined interventions performed on the thoracic spine to improve shoulder functions in patients with SIS. As thoracic kyphosis is related to SIS [3,4], and thoracic kyphosis induces changes in noncontractile and contractile tissues, manual therapy [19] and therapeutic exercise (extension exercise) are required [16,20].

Therefore, this pilot study was performed to investigate the effectiveness of thoracic mobilization and extension exercise individually and in combination in the treatment of kyphosis and shoulder functions of patients with SIS.

## 2. Materials and Methods

### 2.1. Participants

The study population consisted of patients with SIS from the Pain Medicine Clinic in Seoul City, South Korea. Thirty individuals were recruited for the study. The inclusion criteria were: (1) patients diagnosed with SIS; (2) patients with thoracic kyphosis ≥40° [21]; (3) patients who had received at least two positive signs on their Neer sign, Hawkins sign, supraspinatus test, apprehension test, and relocation test [22]; and (4) patients who consented to the purposes of this study and participated voluntarily. The exclusion criteria were: (1) patients with a rotator cuff tear or a previous history of shoulder surgery and (2) patients with a previous history of cervical or thoracic spine surgery.

### 2.2. Study Design

In the present study, 30 patients with SIS were recruited. For patients with SIS, thoracic joint mobilization has an effect size of 0.86 for self-reported pain for shoulder movement [5]. For the sample size of a pilot randomized study, 10 participants per group are recommended for a large (0.8) effect size. Therefore, in the present study, 10 participants per group were included [23]. A randomized controlled trial design was used, and patients were randomly divided into 3 groups: mobilization group (thoracic joint mobilization, *n* = 10), exercise group (extension exercise groups, *n* = 10), or combination group (thoracic joint mobilization and extension exercise group, *n* = 10). During the randomization procedure, all subjects received a numbered card from an administrator who was not involved in the study. Using an online randomization web service (https://www.randomizer.org/) to computerize random numbers, the subjects were divided into three groups based on the number on their cards. Baseline tests were performed prior to intervention, and post-hoc testing was performed after the intervention was completed. All groups completed the intervention for 4 weeks without dropouts. This study was conducted in accordance with the Helsinki Declaration. This study was approved by the Yong-in Institutional Ethical Review Board (ethical approval number 2-1040966-AB-N-01-20-2002-HSR-174-2). Written consent was obtained from all subjects.

### 2.3. Protocol

Exercise programs were supervised by a single physical therapist. Each intervention consisted of 3 sessions per week for 4 weeks (i.e., a total of 12 sessions), with each session lasting around 15 min. For mobilization and exercise, interventions were performed in groups, and all patients were provided with the same amount of interventions. Joint mobilization was performed by a physical therapist who received at least 300 h of manual therapy educational training.

#### 2.3.1. Mobilization Group

Joint mobilization to the thoracic spine consisted of oscillations, performed in the prone positions for 30 repetitions, with 1 min rest between 4 sets. For central posterior-anterior mobilization, a grade III large amplitude rhythmic oscillation was applied to the thoracic spine [8]. The location of application on the thoracic spine was the joint sign segment (the patient’s most painful or stiffest segment) described by Maitland through a passive accessory intervertebral motion (PAIVM) test. If the joint sign was not present, posterior-anterior mobilization was performed on T6-T7 [5]. Joint mobilization was performed for 15 min.

#### 2.3.2. Exercise Group

The exercise programs were designed with an aim to improve thoracic spine extension, trunk extensor muscle strength, and trunk flexor muscle flexibility. Exercise programs consisted of foam roll stretches (warm-up), marching on roller (10 reps × 2), thoracic extension at a wall using bodyweight (10 reps × 2), and standing neck/chest stretch (cool-down) [20,24]. The exercise programs were performed for 15 min.

#### 2.3.3. Combination Group

The intervention of the combination group consisted of joint mobilization plus the exercise program. Joint mobilization consisted of oscillations (central posterior-anterior mobilization) performed in the prone position for 30 repetitions, with 1 min rest between the 2 sets. Additionally, the exercise program was performed with foam roll stretches (warm-up), marching on roller (10 reps × 1), thoracic extension at a wall using bodyweight (10 reps × 1), and standing neck/chest stretch (cool-down). This combination therapy was performed for 15 min (joint mobilization 7 min 30 s + extension exercise 7 min 30 s = 15 min).

### 2.4. Evaluation

All outcomes measured were assessed at baseline and after 4 weeks of intervention. The testers were blind to the purpose of the study. All participants were evaluated by a physiotherapist at the baseline and post-test (after 4 weeks). The main outcome measures were the thoracic kyphosis angle and SPADI total point.

#### 2.4.1. Thoracic Kyphosis

Thoracic kyphosis angle was calculated using an inclinometer (Baseline^®^ bubble inclinometer; Fabrication Enterprises, White Plains, NY, USA). The intraclass correlation coefficients (ICCs) (average) for thoracic kyphosis on the inclinometer ranged from 0.94 to 0.98 [25]. First, an inclinometer was placed over the spinous process of T1 and T2 for measurement (first inclinometer angle). Second, another inclinometer was placed over the spinous process of T12 and L1 for angle measurement (second inclinometer angle). The kyphosis angle was taken as the first inclinometer angle plus the second inclinometer angle [2,25].

#### 2.4.2. Muscles Tone

Muscles tone was measured using the MyotonPro^®^ (Myoton AS, Tallinn, Estonia). The muscles selected in this study were the upper trapezius and pectoralis major. On the MyotonPRO^®^, the ICCs of the upper trapezius and pectoralis major ranged from 0.85 to 0.98 [26]. Prior to measurement, subjects remained at rest for 10 min to eliminate any unnecessary anxiety. The measurements were taken by placing the MyotonPRO^®^ tip vertically in the midpoint of the subjects’ upper trapezius and pectoralis major muscles belly [26]. The changes in tone (frequency, H_Z_) and stiffness (N/m) obtained through the MyotonPRO^®^ were used as the outcomes.

#### 2.4.3. Shoulder Joint ROM Measurements

A goniometer (Goniometer, Sammons Preston, Performance Health Supply Inc., Warrenville, IL, USA) was used for passive shoulder joint ROM measurements. On the goniometer, the ICCs of shoulder joint range of motion measurements ranged from 0.84 to 0.90 [27]. Patients’ passive ROM angles of the affected side shoulder joint (flexion, abduction, medial rotation, and lateral rotation) were measured in a supine position. All measurements were taken within a pain-free range and kept free of compensatory motions.

#### 2.4.4. Shoulder Pain and Disability Index (SPADI)

The SPADI was used for the assessment of shoulder pain and disability. The ICCs from the Korean version of SPADI ranged from 0.98 to 0.99, demonstrating high reliability [28]. SPADI consists of 5 categories of pain and 8 categories of disability. All categories have a visual analog scale ranging from 0 to 10 points. A score of 0 in a pain category indicates that the subject feels no pain, while a score of 10 indicates that the subject is experiencing very severe pain. Likewise, a score of 0 in a disability category indicates no discomfort, whereas a score of 10 indicates unbearable discomfort. Thus, a higher score indicates a greater severity of pain or disability. The subjects were asked to check the SPADI categories based on their current conditions. In this study, the sum of all 13 categories was used as the total outcome.

### 2.5. Statistical Analysis

SPSS 20.0 (SPSS Inc., Chicago, IL, USA) for Windows was used to statistically analyze the effectiveness of thoracic mobilization, extension exercise, and the combination of interventions on improving the thoracic alignment and shoulder function in SIS patients with kyphosis. The data normality was assessed using the Shapiro-Wilks test, and the homogeneity was tested using one-way analysis of variance (ANOVA). The characteristics of the subjects were analyzed using a chi-square test and descriptive statistics. The difference in baseline and after the intervention was analyzed by a paired *t*-test (thoracic kyphosis, muscles tone and stiffness, ROM, and SPADI). The effect size was calculated using G*power software (Heinrich Heine University, Dusseldorf, Germany). The percentage calculation was a computed change from the baseline for each group. The intervention methods effect was analyzed using one-way ANOVA; a post-hoc least significant difference (LSD) test was then performed (3 groups). In this study, the statistical significance was set to be α = 0.05.

## 3. Results

The general characteristics of the subjects did not differ significantly among the mobilization group, exercise group, and combination group (Table 1). All groups showed a significant increase in the kyphosis, UT and PM muscle tone and stiffness, ROM, and SPADI score (Table 2 and Table 3). In addition, the combination group displayed a significant improvement in thoracic kyphosis and UT muscle tone, as well as flexion, medial rotation, and lateral rotation ROM and SPADI score compared to the mobilization group and exercise group (Table 4 and Table 5).

## 4. Discussions

These results confirm that thoracic mobilization and extension exercise can play a key role in the improvement of thoracic alignment and shoulder function in SIS patients. In addition, combining thoracic mobilization with extension exercise is more effective than a single intervention alone for improving thoracic alignment and shoulder function. In the thoracic kyphosis angle, the mobilization group decreased by −7.87% and the exercise group by −6.12%, while the combination group increased the most to −11.28%. In addition, the combination group was −34.29%, which was more significantly improved in SPADI than the other two groups (mobilization group: −23.55% and exercise group: −21.77%). This means that, for patients with SIS, the noncontractile tissue and contractile tissue problems of the thoracic spine are involved in shoulder dysfunction and that both muscle exercise and joint mobilization are required for trunk extension.

Thoracic kyphosis manifests as limitations in thoracic spine motion caused by changes in noncontractile and contractile tissues [12]. In this study, thoracic joint mobilization was applied to improve hypomobility in noncontractile tissues. Of the thoracic mobilization techniques performed in this study, posteroanterior (P-A) mobilization could increase the extension of the segmentals on which manual force was applied [9]. Therefore, P-A mobilization can correct a kyphotic thoracic spine to a neutral position. A neutral position of the thoracic spine alters the scapular kinematic, increasing the ROM strength [29]. Furthermore, scapular correction reduces the upper trapezius muscle activity [30] and increases the lower trapezius activity [31]. Thoracic mobilization can increase the lower trapezius strength [32] by decreasing the upper trapezius muscle tone [33]. Thus, thoracic mobilization can alter scapular kinematics, improving the upper trapezius muscles, pectoralis major tone, shoulder ROM, and SPADI.

The extension exercise was used for contractile tissue flexibility (pectorals, abdominalis, and psoas) and facilitation (dorsal extension muscles) in this study [16,34]. To prevent lumbar spine extension, expiration control was performed during extension to decrease the lumbar load, and verbal commands of the therapists were used to prevent cervical compensation. In females with thoracic kyphosis, thoracic exercise reduced kyphosis and increased the spinal extensor muscle strength and shoulder ROM [35]. In patients with SIS, thoracic extension exercises improved the pain, SPADI, and ROM [18]. Extension-based thoracic mobilization simultaneously reduces kyphosis [14,15,36,37], reduces the scapular forward distance [15], and increases the pectoralis minor muscle length [37]. Thoracic kyphosis is directly correlated with narrow subacromial spaces [3]. Additionally, thoracic kyphosis indirectly causes SIS by reducing shoulder elevation. Therefore, extension exercises reduce kyphosis through pectoral, abdominal, and psoas stretching, as well as dorsal extension muscle facilitation, and improves the upper trapezius muscles, pectoralis major muscle tone, shoulder ROM, and SPADI through scapular realignments.

Among manual therapy to the thoracic spine, thoracic spine thrust manipulation improved self-reported shoulder pain and SPADI [7]. Thoracic spine and rib manipulation significantly improved the shoulder range of motion (flexion, abduction, and rotation) [38]. In addition, cervical and thoracic spine mobilization reduced the upper trapezius muscle tone [33]. In patients with SIS, manual therapy with therapeutic exercise improved the pain and shoulder strength more than single therapeutic exercise [39]. In the present study, the combination group had a significant increase in thoracic kyphosis; UT muscle tone; and flexion, medial rotation, and lateral rotation ROM and SPADI scores than the mobilization group and exercise group. The results of this study are like those found in previous studies.

Therefore, the mechanism by which combining thoracic mobilization with extension exercise improves thoracic kyphosis and shoulder function appears to be multifactorial. Following passive joint mobilization and active extension exercise, facet joint mobility, abdominal muscle stretching, and back muscle strength may have collectively played a role in correcting the thoracic posture and scapular kinetic changes. Such results correspond to study results indicating that joint mobilization and extension exercise is more effective for shoulder function than extension exercise alone [18]. However, no comparison has been made of joint mobilization alone in previous research. In this study, it has been established that joint mobilization and extension exercise combined was more effective than joint mobilization alone. It is presumed that such results were obtained as thoracic kyphosis directly affected patients with SIS. Optimizing the postural kinetic chain through motor control is necessary for thoracic kyphosis patients [16,34]. Additionally, joint capsule and facet joint hypomobility is presented, along with muscle imbalances in thoracic kyphosis. Thus, this study has demonstrated the need for the correctional postural change of the thoracic spine to improve shoulder functions in patients with SIS, as well as joint mobilization and extension exercises to improve the noncontractile and contractile tissues.

The main limitation of this study is that the total sample size was small. Due to this, the results are not representative of all patients with SIS and thoracic kyphosis and should be considered a pilot study. Another limitation is that four-week intervention periods cannot confirm both short-term and long-term effects. Follow-up studies are required to clarify these limitations. On the other hand, this study used thoracic mobilization, instead of the thoracic manipulation demonstrated in a previous study, to examine kyphosis and shoulder functions in patients with SIS. Joint mobilization is relatively safe with low-velocity techniques, rather than high-velocity thrusts, where the patient is not in control [8,40].

## 5. Conclusions

The purpose of this study was to examine the effects of thoracic joint mobilization and extension exercise mono-interventions and a combination intervention on improving thoracic kyphosis and shoulder function in patients with SIS. Both joint mobilization and extension exercise alone are intervention methods that successfully improve thoracic kyphosis and shoulder function. Meanwhile, the combination therapy of joint mobilization and extension exercise successfully increased thoracic kyphosis, as well as all shoulder functions. Therefore, to improve thoracic kyphosis and shoulder functions in patients with SIS, joint mobilization and mobility exercises are required, and combination therapy should be performed.

## Figures and Tables

**Table 1:** Subject characteristics.

Classification	Joint Mobilization Group (*n* = 10)	Exercise Group (*n* = 10)	Combination Group (*n* = 10)
Gender (male/female)	3/7	3/7	3/7
Affected side (left/right)	4/6	6/4	4/6
Age (years) ^a^	49.20 ± 9.48 ^a^	50.90 ± 9.10	50.20 ± 8.99
Height (cm) ^a^	163.20 ± 10.03	165.00 ± 6.32	164.20 ± 6.58
Weight (kg) ^a^	60.90 ± 11.04	66.90 ± 9.64	61.90 ± 10.42

^a^ Values are denoted as mean ± SD.

**Table 2:** Changes in the thoracic kyphosis angle, UT and PM muscles tone, and stiffness before and after intervention.

Measure/Group	Baseline Test ^a^	After-Test ^a^	%	Effect Size	*p*
Thoracic kyphosis angle (°)					
Mobilization group	44.50 ± 2.07 ^a^	41.00 ± 2.36	−7.87	2.78	0.001
Exercise group	44.10 ± 1.85	41.40 ± 2.72	−6.12	1.40	0.002
Combination group	45.20 ± 2.20	40.10 ± 2.23	−11.28	2.97	0.001
UT muscles tone (H_Z_)					
Mobilization group	15.66 ± 1.07	14.50 ± 0.86	−7.41	2.00	0.001
Exercise group	15.46 ± 1.09	14.40 ± 1.39	−6.86	1.58	0.001
Combination group	15.54 ± 0.87	13.71 ± 1.58	−11.78	2.15	0.001
UT muscles stiffness (N/m)					
Mobilization group	257.90 ± 29.03	232.50 ± 20.49	−9.85	1.89	0.001
Exercise group	257.70 ± 19.33	236.10 ± 27.27	−8.38	1.68	0.001
Combination group	257.50 ± 25.61	223.00 ± 32.83	−13.40	2.32	0.001
PM muscles tone (H_Z_)					
Mobilization group	14.50 ± 1.36	13.72 ± 1.27	−5.38	2.44	0.001
Exercise group	14.26 ± 1.02	13.50 ± 1.18	−5.33	1.90	0.001
Combination group	14.31 ± 1.52	13.43 ± 1.69	−6.15	3.38	0.001
PM muscles stiffness (N/m)					
Mobilization group	229.90 ± 27.18	212.70 ± 25.63	−7.48	2.97	0.001
Exercise group	226.80 ± 21.73	211.40 ± 22.32	−6.79	2.25	0.001
Combination group	224.40 ± 31.22	207.20 ± 33.74	−7.66	3.12	0.001

^a^ Values are means ± SD. UT: upper trapezius and PM: pectoralis major.

**Table 3:** Changes in the affected side shoulder ROM and SPADI point before and after intervention.

Measure/Group	Baseline Test	After-Test	%	Effect Size	*p*
Affected side shoulder flexion ROM (°)					
Mobilization group	138.40 ± 19.31	146.20 ± 20.65	5.64	2.01	0.001
Exercise group	140.60 ± 16.57	148.50 ± 16.24	5.62	1.85	0.001
Combination group	133.80 ± 16.40	146.50 ± 15.91	9.49	2.76	0.001
Affected side shoulder abduction ROM (°)					
Mobilization group	104.60 ± 14.05	112.40 ± 14.88	7.46	2.28	0.001
Exercise group	106.90 ± 9.01	114.30 ± 10.31	6.92	1.77	0.001
Combination group	111.70 ± 19.32	122.30 ± 19.80	9.49	3.03	0.001
Affected side shoulder medial rotation ROM (°)					
Mobilization group	48.00 ± 9.59	50.50 ± 9.34	5.21	1.28	0.003
Exercise group	51.20 ± 10.51	54.10 ± 10.66	5.66	1.27	0.003
Combination group	42.40 ± 14.36	47.60 ± 15.22	12.26	2.10	0.001
Affected side shoulder lateral rotation ROM (°)					
Mobilization group	70.30 ± 7.13	72.50 ± 6.85	3.13	1.05	0.009
Exercise group	69.60 ± 7.46	72.10 ± 8.91	3.59	0.98	0.013
Combination group	62.40 ± 8.77	67.90 ± 9.65	8.81	1.70	0.001
SPADI—pain (point)					
Mobilization group	51.60 ± 12.47	38.60 ± 11.35	−25.19	4.81	0.001
Exercise group	54.00 ± 12.65	40.20 ± 12.84	−25.55	5.37	0.001
Combination group	50.20 ± 10.43	31.60 ± 8.32	−37.05	4.65	0.001
SPADI—disability (point)					
Mobilization group	52.13 ± 15.75	40.38 ± 13.79	−22.54	2.87	0.001
Exercise group	53.50 ± 15.75	43.13 ± 15.45	−19.38	3.60	0.001
Combination group	51.38 ± 14.54	34.63 ± 10.21	−32.60	3.00	0.001
SPADI—total (point)					
Mobilization group	51.92 ± 14.47	39.69 ± 12.81	−23.55	3.59	0.001
Exercise group	53.69 ± 14.51	42.00 ± 14.35	−21.77	4.62	0.001
Combination group	50.92 ± 12.86	33.46 ± 9.39	−34.29	3.83	0.001

ROM: range of motion and SPADI: shoulder pain and disability index.

**Table 4:** Comparison of the thoracic kyphosis angle, UT and PM muscles tone, and stiffness in three intervention groups.

Measure/Group	Value Difference	*F*	*p*	Post-Hoc (LSD)
Thoracic kyphosis angle (°)				
Mobilization group	−3.5 ± 1.26 ^a^	5.340	0.011 *	C > A, B
Exercise group	−2.7 ± 1.94			
Combination group	−5.10 ± 1.72			
UT muscles tone (H_Z_)				
Mobilization group	−1.16 ± 0.58	3.463	0.046 *	C > A, B
Exercise group	−1.06 ± 0.67			
Combination group	−1.83 ± 0.85			
UT muscles stiffness (N/m)				
Mobilization group	−25.40 ± 13.42	2.319	0.118	
Exercise group	−21.60 ± 12.89			
Combination group	−34.50 ± 14.89			
PM muscles tone (H_Z_)				
Mobilization group	−0.78 ± 0.32	0.358	0.702	
Exercise group	−0.76 ± 0.40			
Combination group	−0.88 ± 0.26			
PM muscles stiffness (N/m)				
Mobilization group	−17.20 ± 5.80	0.292	0.749	
Exercise group	−15.40 ± 6.85			
Combination group	−17.20 ± 5.51			

^a^ Values are means ± SD. LSD: least significant difference. * *p* < 0.05.

**Table 5:** Comparison of the affected side shoulder ROM and SPADI point in three intervention groups.

Measure/Group	Value Difference	*F*	*p*	Post-Hoc (LSD)
Affected side shoulder flexion ROM (°)				
Mobilization group	7.80 ± 3.88	4.264	0.025 *	C > A, B
Exercise group	7.90 ± 4.28			
Combination group	12.70 ± 4.66			
Affected side shoulder abduction ROM (°)				
Mobilization group	7.80 ± 3.42	2.192	0.131	
Exercise group	7.40 ± 4.19			
Combination group	10.60 ± 3.50			
Affected side shoulder medial rotation ROM (°)				
Mobilization group	2.50 ± 1.95	4.185	0.026 *	C > A, B
Exercise group	2.90 ± 2.28			
Combination group	5.20 ± 2.48			
Affected side shoulder lateral rotation ROM (°)				
Mobilization group	2.20 ± 2.09	4.668	0.018 *	C > A, B
Exercise group	2.50 ± 2.54			
Combination group	5.50 ± 3.24			
SPADI—pain (point)				
Mobilization group	−13.00 ± 2.70	9.173	0.001 **	C > A, B
Exercise group	−13.80 ± 2.57			
Combination group	−18.60 ± 4.00			
SPADI—disability (point)				
Mobilization group	−11.75 ± 4.09	5.986	0.007 **	C > A, B
Exercise group	−10.37 ± 2.88			
Combination group	−16.75 ± 5.59			
SPADI—total (point)				
Mobilization group	−12.23 ± 3.41	7.846	0.002 **	C > A, B
Exercise group	−11.69 ± 2.53			
Combination group	−17.46 ± 4.55			

^a^ Values are means ± SD. LSD: least significant difference. * *p* < 0.05, ** *p* < 0.01.
